# CC Chemokine Ligand 7 Derived from Cancer-Stimulated Macrophages Promotes Ovarian Cancer Cell Invasion

**DOI:** 10.3390/cancers13112745

**Published:** 2021-06-01

**Authors:** Miran Jeong, Yi-Yue Wang, Ju-Yeon Choi, Myong Cheol Lim, Jung-Hye Choi

**Affiliations:** 1 Division of Molecular Biology, College of Pharmacy, Kyung Hee University, Seoul 02447, Korea; jeongmr@khu.ac.kr; 2 Department of Life and Nanopharmaceutical Science, Kyung Hee University, Seoul 02447, Korea; wangyiyue@khu.ac.kr (Y.-Y.W.); chlwndus001@khu.ac.kr (J.-Y.C.); 3 Division of Tumor Immunology, Center for Gynecologic Cancer & Center for Clinical Trials, National Cancer Center, Goyang 10408, Korea; mclim@ncc.re.kr

**Keywords:** CCL7, CCR3, tumor-associated macrophages, ovarian cancer

## Abstract

**Simple Summary:**

Ovarian cancer is the most lethal of gynecological malignancies. Although metastasis leads to a bleak prognosis and high mortality, the molecular and cellular processes of ovarian cancer metastasis remain largely unclear. Non-malignant cells including macrophages constitute a unique microenvironment that can completely change the nature of malignant cells at various levels. In this study, we found that the expression of chemokine (C-C motif) ligand 7 (CCL7), a soluble inflammatory factor, was enhanced in macrophages stimulated by human ovarian cancer cells as well as peritoneal macrophages from patients with ovarian cancer; CCL7 promoted the invasion of ovarian cancer cells. Our study elucidates the crosstalk between macrophages and ovarian cancer cells that contributes to ovarian cancer metastasis.

**Abstract:**

In the tumor microenvironment, macrophages have been suggested to be stimulated by tumor cells, becoming tumor-associated macrophages that promote cancer development and progression. We examined the effect of these macrophages on human ovarian cancer cell invasion and found that conditioned medium of macrophages stimulated by ovarian cancer cells (OC-MQs) significantly increased cell invasion. CC chemokine ligand 7 (CCL7) expression and production were significantly higher in OC-MQs than in the control macrophages. Peritoneal macrophages from patients with ovarian cancer showed higher CCL7 expression levels than those from healthy controls. Inhibition of CCL7 using siRNA and neutralizing antibodies reduced the OC-MQ-CM-induced ovarian cancer cell invasion. CC chemokine receptor 3 (CCR3) was highly expressed in human ovarian cancer cells, and a specific inhibitor of this receptor reduced the OC-MQ-CM-induced invasion. Specific signaling and transcription factors were associated with enhanced CCL7 expression in OC-MQs. CCL7-induced invasion required the expression of matrix metalloproteinase 9 via activation of extracellular signal-related kinase signaling in human ovarian cancer cells. These data suggest that tumor-associated macrophages can affect human ovarian cancer metastasis via the CCL7/CCR3 axis.

## 1. Introduction

Ovarian cancer is the leading cause of death among all gynecological cancers and is the seventh most fatal of all cancers in women worldwide, responsible for approximately 184,000 deaths in 2018 [1]. The disease has subtle symptoms at early stages, and the majority of ovarian cancer cases are diagnosed at an advanced stage characterized by extensive invasion and metastasis. Patients with advanced stages (Stage III and IV) have a poor prognosis and high mortality. In this regard, the 5 year survival rate of patients with ovarian cancer remains extremely low (less than 40%), despite recent advances in treatment [2,3]. Although metastasis leads to a bleak prognosis and high mortality, the molecular and cellular processes of ovarian cancer metastasis remain largely unclear. 

Distinct from other hematogenously metastasizing cancers, ovarian cancer is associated with peritoneal dissemination [4,5], and numerous studies have suggested the involvement of the intraperitoneal tumor microenvironment [6,7,8]. The tumor microenvironment consists of cancer cells and a variety of non-malignant cells including endothelial cells, stromal fibroblasts, adipocytes, and immune cells (lymphocytes and macrophages) [9]. Non-malignant cells constitute a unique microenvironment that can completely change the nature of malignant cells at various levels [10]. Among the non-malignant cells, tumor-associated macrophages (TAMs) are one of the most abundant cell types and they have been studied extensively for their pro-tumoral activity [11,12,13,14]. In ovarian cancer, macrophage density has a significant positive correlation with cancer stage and grade [15]. In addition, TAM infiltration is associated with a poor prognosis in patients with ovarian cancer [16].

The potential role of soluble inflammatory factors in the tumor microenvironment, particularly those from TAMs, has been suggested to contribute to the lethal metastatic nature of cancer in humans [17,18,19,20,21]. For example, the inflammatory cytokines derived from macrophages, colony-stimulating factor-1 and interleukin (IL)-17, promote the invasion of breast carcinoma cells [20,21], and TAMs stimulate the metastatic potential of thyroid papillary cancer by releasing IL-8 [22]. However, the role of inflammatory factors in ovarian cancer progression remains poorly understood. 

In this study, we found that the expression of chemokine (C-C motif) ligand 7 (CCL7), also known as monocyte chemotactic protein 3, was enhanced in macrophages stimulated by human ovarian cancer cells as well as peritoneal macrophages from patients with ovarian cancer; CCL7 promoted the invasion of ovarian cancer cells. Our study elucidates the crosstalk between macrophages and ovarian cancer cells that contributes to ovarian cancer metastasis. 

## 2. Results

### 2.1. Macrophages Stimulated by Ovarian Cancer Cells Induce Cancer Cell Invasion, and Show Enhanced Expression and Production of ccl7

**Figure 1:**
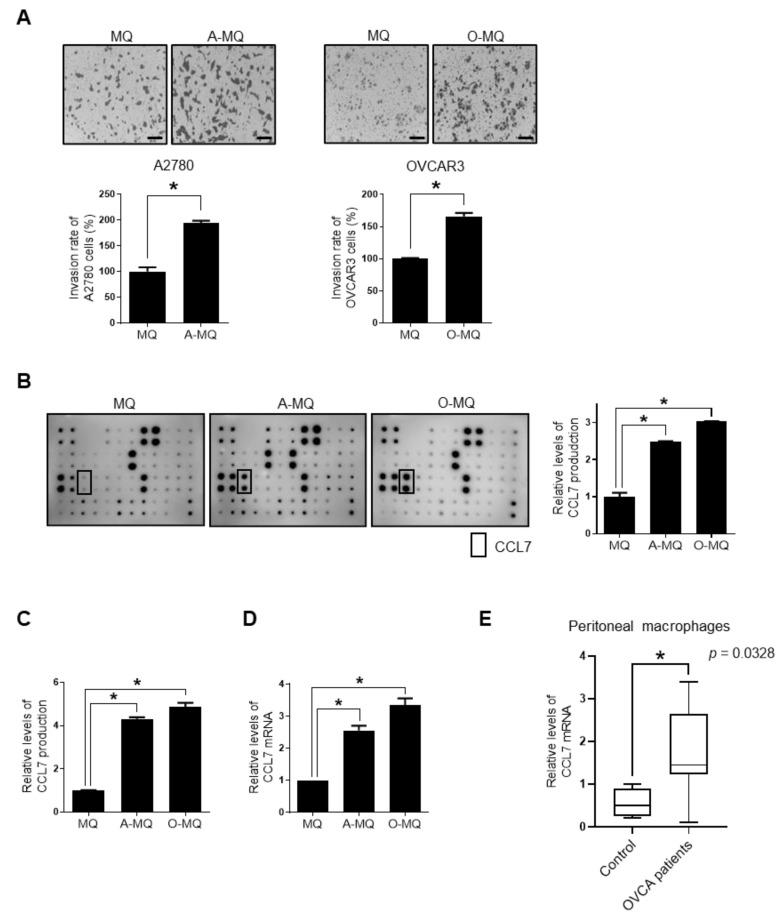
Increased expression of CC chemokine ligand 7 (CCL7) in macrophages stimulated by ovarian cancer cells. (**A**) Human ovarian A2780 and OVCAR3 cancer cells were resuspended with the conditioned medium of macrophages (MQ), macrophages stimulated by A2780 cells (A-MQ), and macrophages stimulated by OVCAR3 cells (O-MQ), and seeded in Matrigel-coated upper chambers. The cells were allowed to invade for 48 h. Scale bar, 100 µm. (**B**) A cytokine antibody array was used to analyze the cytokine profiles of the conditioned medium of MQ, A-MQ, and O-MQ. Values from the cytokine array membrane were evaluated by densitometry. (**C**) CCL7 production from MQ, A-MQ, and O-MQ was analyzed by ELISA kits. (**D**) The expression of CCL7 in MQ, A-MQ, and O-MQ was measured by the real-time polymerase chain reaction. (**E**) The expression of CCL7 in peritoneal macrophages of women with non-malignant tumors (control) and advanced ovarian cancer (OVCA). * *p* < 0.05.

To explore whether soluble factors from TAMs in the peritoneal microenvironment contributed to the invasiveness of ovarian cancer cells, CM of macrophages stimulated by ovarian cancer A2780 and OVCAR3 cells (A-MQ and O-MQ, respectively) were used in this study. The CM significantly enhanced the invasion of ovarian cancer cells (Figure 1A). 

TAMs are known to secrete numerous cytokines, inflammatory factors, and growth factors that are implicated in tumor metastasis [23]. A human cytokine antibody array was used for profiling ovarian cancer-stimulated macrophages (OC-MQ) to identify the factors that stimulate cell invasion. Both A-MQ and O-MQ produced higher amounts of CCL7 than macrophages without cancer cell stimulation (Figure 1B). Enhanced secretion of CCL7 in OC-MQ was further quantified using an enzyme-linked immunoassay kit (Figure 1C). A-MQ and O-MQ increased the expression level of CCL7 mRNA, suggesting that enhanced levels of CCL7 production from OC-MQ are associated with increased transcription of the gene (Figure 1D). To verify the clinical significance of CCL7, its expression was investigated in peritoneal macrophages from patients with ovarian cancer using publicly available datasets (Figure 1E). Similar to A-MQ and O-MQ, these macrophages expressed higher levels of CCL7 compared to those from healthy controls. Together, these data suggest that macrophages stimulated by cancer cells produce high levels of CCL7, which can play a role in human ovarian cancer invasion. 

### 2.2. CCL7 Derived from OC-MQ Promotes Ovarian Cancer Invasion through CCR3

**Figure 2:**
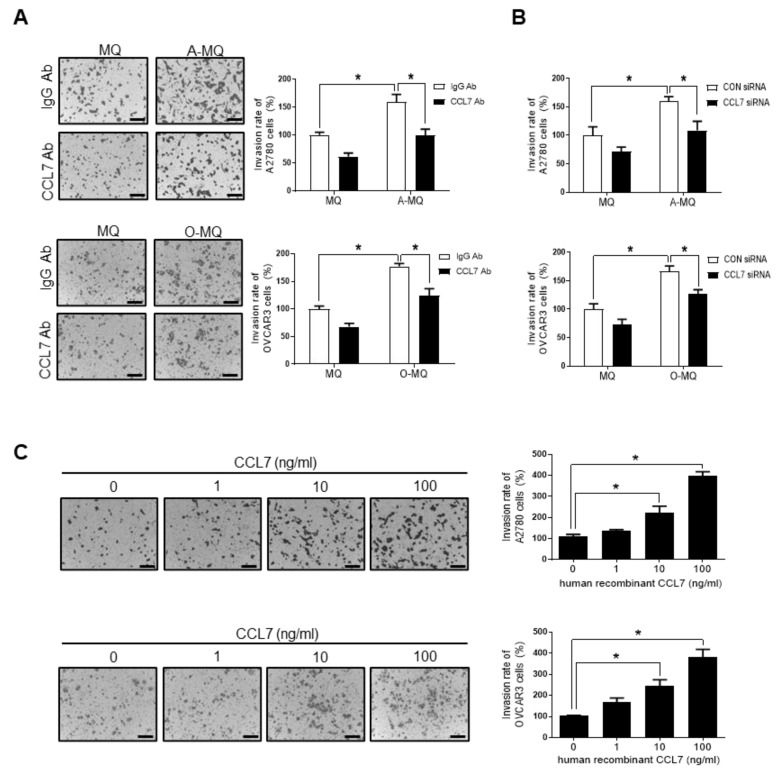
Involvement of CC chemokine ligand 7 (CCL7) in ovarian cancer cell invasion induced by ovarian cancer-stimulated macrophages (OC-MQ). (**A**) Human ovarian cancer A2780 and OVCAR3 cells were resuspended with the conditioned medium of macrophages (MQ), macrophages stimulated by A2780 cells (A-MQ), and macrophages stimulated by OVCAR3 cells (O-MQ) in the absence or presence of a CCL7 neutralizing antibody (3 µg/mL), and seeded in Matrigel-coated upper chambers. The ovarian cancer cells were allowed to invade for 48 h. Scale bar, 100 µm. (**B**) A2780 and OVCAR3 cells were resuspended with the conditioned medium of MQ, A-MQ, and O-MQ transfected with control siRNA or a specific CCL7 siRNA (50 nM) for 24 h. The cells were seeded in Matrigel-coated upper chambers. The A2780 and OVCAR3 cells were allowed to invade for 48 h. (**C**) A2780 and OVCAR3 cells were treated with human recombinant CCL7 (0, 1, 10, 100 ng/mL) for 24 h and seeded in Matrigel-coated upper chambers. The cells were allowed to invade for 48 h. Scale bar, 100 µm. * *p* < 0.05.

We investigated whether CCL7 secreted from OC-MQ directly contributed to the invasion of human ovarian cancer cells. Neutralizing anti-CCL7 antibodies significantly suppressed the invasion of ovarian cancer cells stimulated by A-MQ and O-MQ (Figure 2A). Similarly, knockdown of CCL7 in macrophages using a specific siRNA resulted in significant suppression of ovarian cancer cell invasion (Figure 2B). Moreover, treatment with human recombinant CCL7 markedly increased the invasion in a concentration-dependent manner (Figure 2C). These observations indicate that OC-MQ-stimulated ovarian cancer cell invasion is associated with enhanced levels of CCL7 in macrophages.

**Figure 3:**
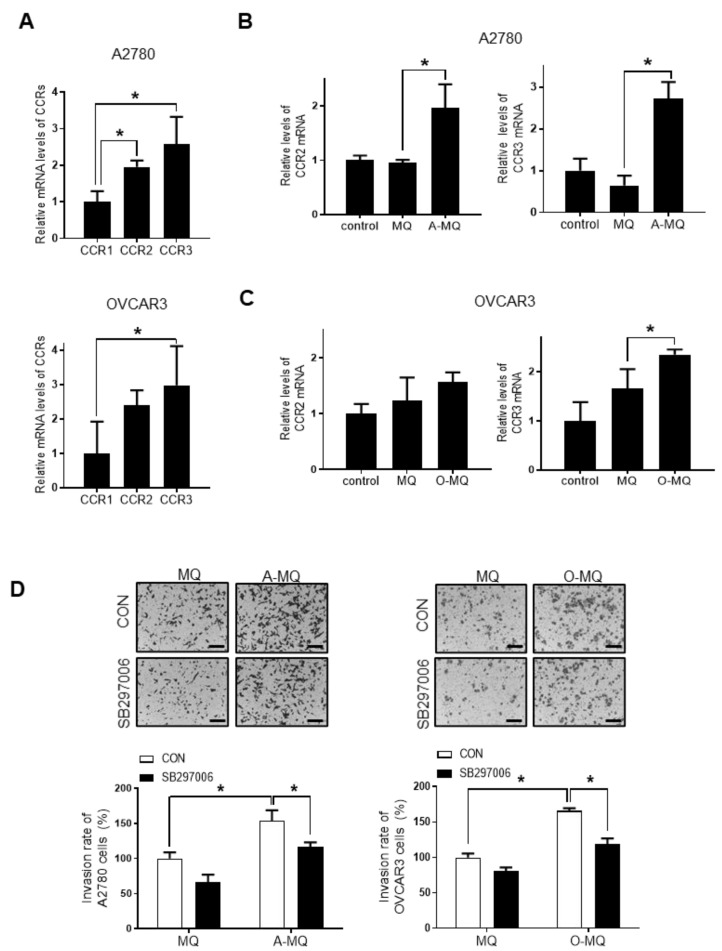
Involvement of CC chemokine receptor 3 (CCR3) in CC chemokine ligand 7 (CCL7)-induced ovarian cancer cell invasion. (**A**) The mRNA levels of CCR1, CCR2, and CCR3 in A2780 and OVCAR3 cells were measured by the real-time polymerase chain reaction (RT-PCR). (**B**) A2780 cells were treated with the conditioned medium of macrophages (MQ) and macrophages stimulated by A2780 cells (A-MQ). The mRNA levels of CCR2 and CCR3 in A2780 cells were detected by RT-PCR. (**C**) OVCAR3 cells were treated with the conditioned medium of MQ and macrophages stimulated by OVCAR3 cells (O-MQ). (**D**) A2780 and OVCAR3 cells were resuspended with the CM of MQ, A-MQ, and O-MQ following pretreatment with the CCR3 inhibitor, SB297006 (20 µM), for 1 h. The cancer cells were seeded in Matrigel-coated upper chambers and allowed to migrate for 48 h. Scale bar, 100 µm. * *p* < 0.05.

All three types of CC chemokine receptors, CCR1, CCR2, and CCR3, were expressed in both A2780 and OVCAR3 cells, though CCR1 showed relatively low expression compared with the other two receptors (Figure 3A); the levels of CCR3 were the highest. A-MQ stimulated CCR2 expression in A2780 cells but not in OVCAR3 cells (Figure 3B). In addition, the CCR2 inhibitor did not affect OC-MQ-stimulated invasion of ovarian cancer cells (data not shown). In contrast, when A2780 and OVCAR3 cells were stimulated by OC-MQ, they both showed increases in CCR3 expression levels (Figure 3B,C). Moreover, OC-MQ-stimulated ovarian cancer cell invasion was markedly attenuated by SB297006, a specific CCR3 inhibitor (Figure 3D). These findings suggest that CCR3 acts as a key receptor for CCL7-mediated ovarian cancer invasion.

### 2.3. PHF8 Is Involved in Upregulating CCL7 in OC-MQ

**Figure 4:**
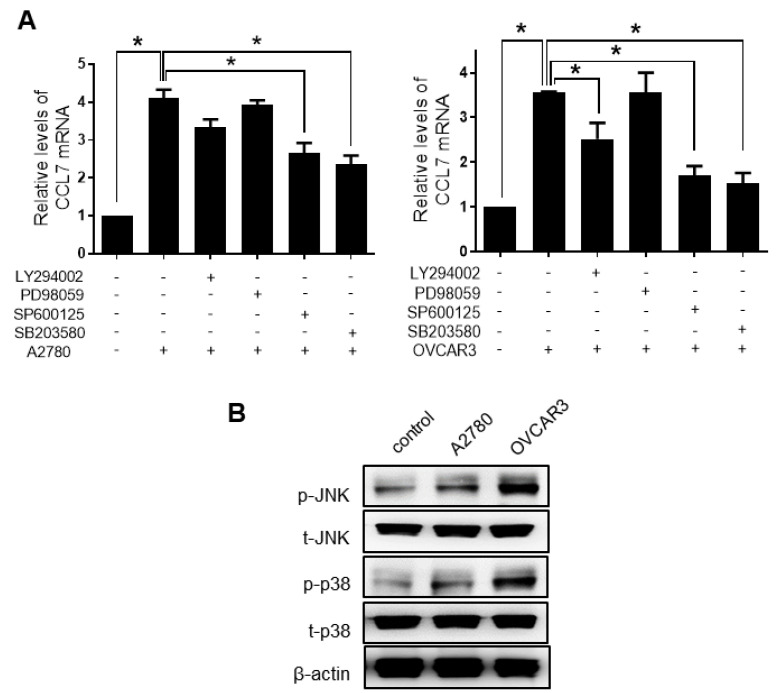
Involvement of p38/c-Jun N-terminal kinase (JNK) signaling in CC chemokine ligand 7 (CCL7) expression in macrophages stimulated by ovarian cancer cells. (**A**) Macrophages were pretreated with PI3K inhibitor, LY294002 (20 µM), MAPK/ERK Kinase (MEK) inhibitor, PD98059 (70 µM), JNK inhibitor, SP600125 (20 µM), or p38 inhibitor, SB203580 (20 µM), for 1 h, then treated with the CM of A2780 and OVCAR3 cells for 24 h. The mRNA levels of CCL7 were measured by the real-time polymerase chain reaction. (**B**) The levels of phosphorylated JNK, total JNK, phosphorylated p38, and total p38 in macrophages (MQ), macrophages stimulated by A2780 cells (A-MQ), and macrophages stimulated by OVCAR3 cells (O-MQ) were analyzed by Western blot assays. Original Western Blots shown in Figure S6. * *p* < 0.05.

According to previous reports, the expression of CCL7 can be regulated by several signaling pathways including PI3K/Akt, JNK, ERK1/2, and p38 [24,25]. To verify which signaling pathway was involved in the upregulation of CCL7 in OC-MQ, specific inhibitors were used. The upregulation of CCL7 in macrophages stimulated with the CM of ovarian cancer cells (both A2780-CM and OVCAR3-CM) was significantly negated by the p38 inhibitor, SB203580, and the JNK inhibitor, SP600125 (Figure 4A). The PI3K inhibitor LY294002 significantly reduced the CCL7 upregulation by OVCAR3-CM, but not that by A2780-CM. PD98059, a MAPK/ERK Kinase (MEK) inhibitor, did not significantly impact the CM stimulation, suggesting that the MEK/ERK pathway is not associated with the CCL7 upregulation. Additionally, the CM of ovarian cancer activated the p38 and JNK pathways in macrophages (Figure 4B). These data suggest that the p38/JNK signaling pathway may be associated with upregulating CCL7 in OC-MQ.

**Figure 5:**
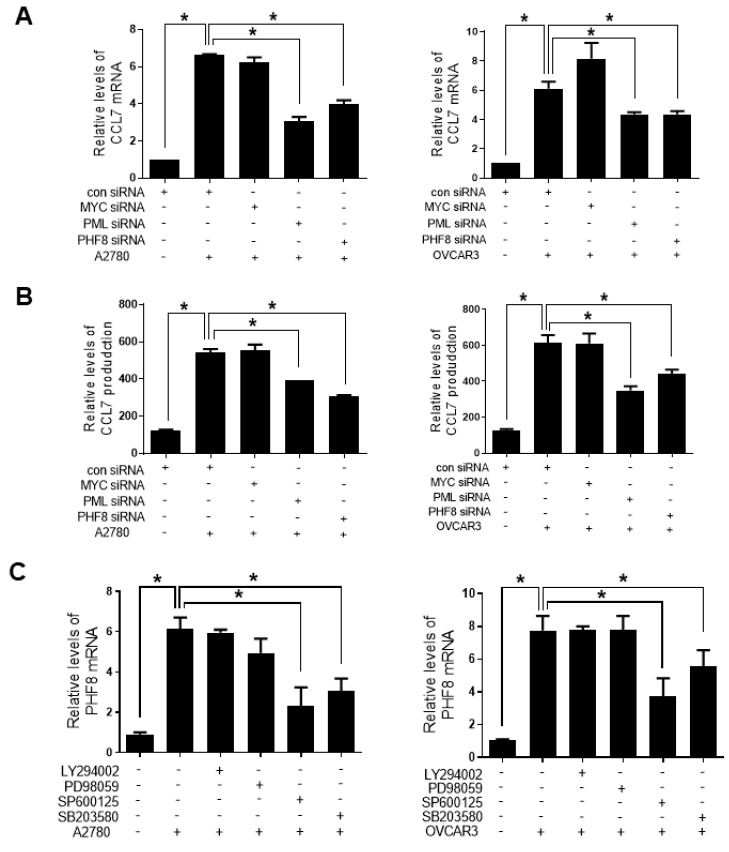
Involvement of PHF8 in CC chemokine ligand 7 (CCL7) expression in macrophages stimulated by ovarian cancer cells. (**A**,**B**) Macrophages were transfected with MYC, PML, or PHF8 siRNA (50 nM), or control siRNA, then treated with the conditioned medium (CM) of A2780 and OVCAR3 cells for 24 h. (**A**) The mRNA levels of CCL7 were measured by the real-time polymerase chain reaction. (**B**) The levels of CCL7 in the medium of macrophages were measured by an enzyme-linked immunoassay kit. (**C**) Macrophages were pretreated with the PI3K inhibitor, LY294002 (20 µM), MAPK/ERK Kinase (MEK) inhibitor, PD98059 (70 µM), JNK inhibitor, SP600125 (20 µM), or p38 inhibitor, SB203580 (20 µM), for 1 h, then with the CM of A2780 and OVCAR3 cells for 24 h. The mRNA levels of PHF8 were measured by the RT-PCR. * *p* < 0.05.

Because MYC, PML, and PHF8 have been implicated in human cancer metastasis [26,27,28], we investigated whether they were involved in CCL7 upregulation in OC-MQ. PML and PHF8 knockdown, but not MYC knockdown, significantly decreased the expression and production of CCL7 in macrophages stimulated by both A2780 and OVCAR3 (Figure 5A,B). Additionally, the expression of PHF8 mRNA in macrophages was suppressed by both p38 and JNK inhibitors (Figure 5C). In contrast, neither the p38 nor the JNK inhibitor affected A2780-CM-induced PML expression in macrophages (data not shown). These findings suggest that PHF8 is involved in regulating CCL7 via the p38/JNK pathway in OC-MQ.

### 2.4. ERK Signaling and MMP Expression Are Required for CCL7-induced Ovarian Cancer Cell Invasion

**Figure 6:**
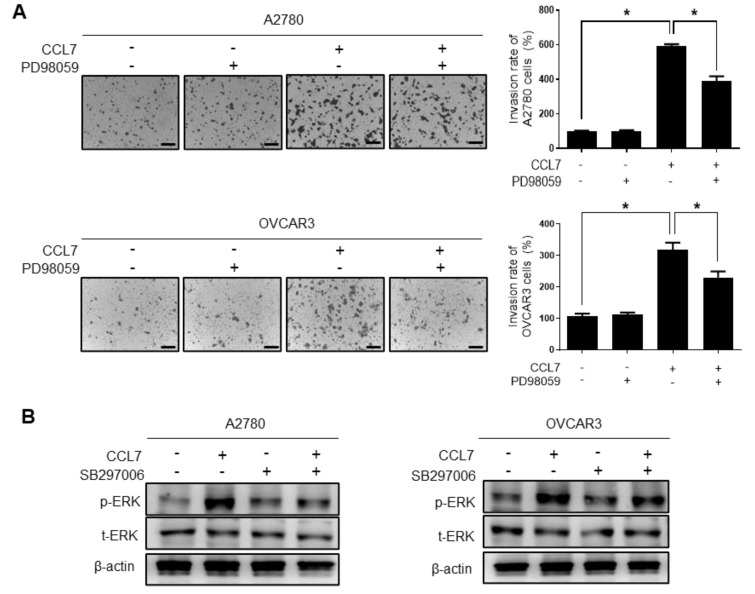
Involvement of extracellular signal-regulated kinase (ERK) signaling in CC chemokine ligand 7 (CCL7)-induced ovarian cancer cell invasion. (**A**) A2780 and OVCAR3 cells were pretreated with the MAPK/ERK Kinase (MEK) inhibitor, PD98059 (70 µM), for 1 h, then treated with recombinant CCL7 (100 ng/mL) for 24 h. The cancer cells were seeded in Matrigel-coated upper chambers and allowed to migrate for 48 h. Scale bar, 100 µm. (**B**) A2780 and OVCAR3 cells were pretreated with the CCR3 inhibitor, SB297006 (20 µM), for 1 h, then with recombinant CCL7 (100 ng/mL) for 24 h. The levels of phosphorylated and total ERK in A2780 and OVCAR3 cells were analyzed by Western blot assays. Original Western Blots shown in Figure S6. * *p* < 0.05.

**Figure 7:**
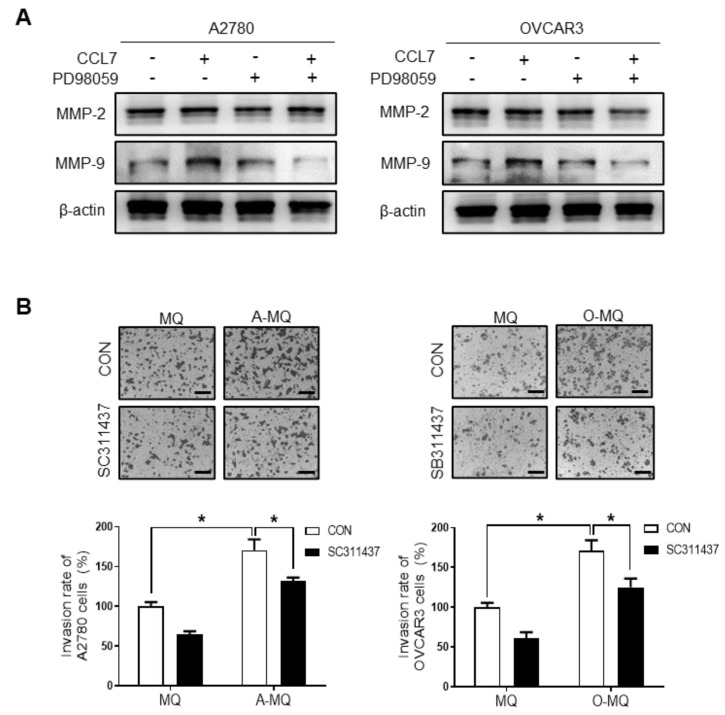
Involvement of matrix metalloproteinase (MMP)-9 in CC chemokine ligand 7 (CCL7)-induced ovarian cancer cell invasion. (**A**) A2780 and OVCAR3 cells were pretreated with the MAPK/ERK Kinase (MEK) inhibitor, PD98059 (70 µM), for 1 h, then with recombinant CCL7 (100 ng/mL) for 24 h. The expression of MMP-2 and MMP-9 in A2780 and OVCAR3 cells was detected by Western blot assays. (**B**) A2780 and OVCAR3 cells were pretreated with the MMP-9 inhibitor, SC311437 (20 µM), for 1 h, then with the conditioned medium of macrophages (MQ), macrophages stimulated by A2780 cells (A-MQ), and macrophages stimulated by OVCAR3 cells (O-MQ) for 24 h. The cancer cells were seeded in Matrigel-coated upper chambers and allowed to migrate for 48 h. Scale bar, 100 µm. The results represent the means ± SD of at least three independent experiments. Original Western Blots shown in Figure S6. * *p* < 0.05.

Given that the ERK signaling pathway is associated with the migration and invasion of malignant tumors [29] and CCL7-associated pro-cancer activities [30], we investigated the role of ERK signaling in CCL7-induced ovarian cancer cell invasion. As shown in Figure 6A, the CCL7-stimulated increase in ovarian cancer cell invasion was significantly reduced in the presence of PD98059. We then investigated whether the activation of ERK signaling was affected by CCL7 binding to CCR3; ERK was activated by CCL7 treatment, and the CCR3 inhibitor, SB297006, hindered ERK activation in A2780 and OVCAR3 cells (Figure 6B). We further evaluated the effect of ERK activation by CCL7 on the expression of MMP-2 and -9, which play critical roles in ovarian cancer metastasis. MMP-9 expression was significantly increased by CCL7 treatment in A2780 and OVCAR3 cells, and PD98059 markedly reduced the expression of MMP-9, but not MMP-2, another key gelatinase (Figure 7A). Moreover, SC311437, a MMP-9 inhibitor, significantly suppressed the OC-MQ-induced invasion of A2780 and OVCAR3 cells (Figure 7B). Together, these data indicate that CCL7 overproduction from macrophages stimulated by cancer cells increases the invasion of ovarian cancer cells by regulating MMP-9 via the ERK pathway. 

## 3. Discussion

There are a large number of macrophages in the ascites of patients with late stages of ovarian cancer, and these macrophages are involved in tumor progression by modifying the tumor microenvironment [31,32]. In this study, the CM of ovarian cancer-stimulated macrophages (OC-MQ, A-MQ, and O-MQ) that contained various soluble factors produced by macrophages markedly increased the invasiveness of ovarian cancer cells. These data are consistent with previous findings showing the potential role of TAMs in ovarian cancer metastasis [32,33,34]. 

Enhanced inflammation accelerated the development of ascites and metastases, and depletion of peritoneal macrophages reduced metastasis, in an ovarian cancer mouse model [32]. The CM of ascites-derived TAMs increased the migration of cultured patient-derived high-grade serous ovarian cancer cells [33]. Notably, a number of studies have suggested that the peritoneal macrophages of patients with ovarian cancer show an M2 phenotype with pro-cancer activities [35,36,37]. Here, we found that both the A-MQ and O-MQ used in this study enhanced the expression of M2 phenotype markers such as CD206 and TREM (Supplementary Figure S1). These data suggest that macrophages may be alternatively activated (M2 polarization) by stimulation with ovarian cancer cells, which is critical for macrophage-stimulated invasion of ovarian cancer cells. However, considering the limitations of PMA-differentiated THP-1 macrophages used in this study, further study using peritoneal macrophages from patients with ovarian cancer is needed in order to strengthen our findings.

Chemokines play a key role in various aspects of cancer development and progression [38]. They are formed by various cell types and classified into CC, CXC, CX3C, and C subfamilies according to the number and array of conserved cysteines. We found that OC-MQ produced a large amount of the CC subfamily chemokine, CCL7, compared to control macrophages, and the levels of CCL7 were significantly enhanced in peritoneal macrophages of patients with ovarian cancer. Considering other CC subfamily chemokines, CCL2 and CCL5 have also been implicated in the progression of a wide range of cancers. We examined their levels in the peritoneal macrophages and found that they were not significantly different from those in the control groups (Supplementary Figure S2). In addition, IOSE80PC-MQ, from macrophages stimulated by normal ovarian surface epithelial cells (IOSE80PC), showed no increase in CCL7 production or mRNA expression (Supplementary Figure S3), indicating that macrophages stimulated by ovarian cancer cells, but not their normal counterpart, can highly produce CCL7.

CCL7, a chemokine that attracts various leukocytes, has been implicated in some immune disorders [39], and a potential role of CCL7 in cancer has been suggested [39]. Although some reports have shown the anti-tumor activity of CCL7 [40], most studies support its pro-tumor effects [39]. Some cancer cells, including stomach [41], colon [42], prostate [43], and renal cells, show an abnormal increase in CCL7 expression levels, which is associated with tumor progression [44]. Furthermore, CCL7 levels were higher in liver metastasis of colon cancer than in primary colon cancer [42], and this CCL7 overexpression increased cell growth, migration, and invasion in in vitro and in vivo models [45]. In addition to cancer cells, non-malignant cells in the tumor microenvironment, including fibroblasts, adipocytes, and macrophages, were found to produce CCL7 [39]. CCL7 derived from cancer-associated fibroblasts stimulated the migration and invasion of oral squamous cell carcinoma [46,47] and liver cancer [48]. In this study, we demonstrated that CCL7 from macrophages stimulated by ovarian cancer cells increased ovarian cancer cell invasion and migration (Supplementary Figure S4), suggesting that TAM-derived CCL7 is a key factor for ovarian cancer metastasis. To the best of our knowledge, this is the first study to show a potential role of CCL7 in human ovarian cancer.

Chemokines function by binding to their receptors expressed on various kinds of responsive cells. CCR1, CCR2, and CCR3 are widely considered the main receptors of CCL7 [49]. These receptors have been implicated in several diseases, including cancer [50,51,52]. For example, CCR1 expression in cancer cells is critical for fibroblasts to stimulate breast cancer cell proliferation [53], CCR2 exhibited a pro-tumor effect by stimulating the recruitment of monocytes/macrophages in lymphoma, melanoma, and breast cancer [54,55], and CCR3 was overexpressed in malignant cutaneous tumors, including malignant melanomas [56]. CCR3 expression in tumor tissues has been also shown to correlate with the grade of malignancy in human renal cancer [57]. In addition, CCR3 is associated with invasion of prostate cancer cells [58]. It has been reported that CCL5-induced invasion of ovarian cancer stem-like cells is mediated by CCR1 and CCR3 [59], and CCR3, along with CCR2 and CCR5, are associated with CCL11-stimulated proliferation, migration, and invasion of ovarian cancer cells [60]. In the current study, we demonstrated that CCL7 derived from macrophages stimulated the invasion and migration of ovarian cancer cells via CCR3. Together, these data suggest that CCR3 in human ovarian cancer cells can be stimulated by CCL7 from TAMs, as well as CCL5 and CCL11, and can play a critical role in ovarian cancer metastasis.

CCL7 was upregulated in rat astrocytes via stimulation of the mitogen-activated protein kinase pathway [24]. In primary microglial cells and the Ra2 cell line, Abeta1-42 induced the expression of CCL7 mRNA through the PI3K/Akt signaling pathways [61]. Here, we found that CCL7 upregulation in macrophages stimulated by the CM of ovarian cancer cells was associated with the p38/JNK pathway. Moreover, the p38/JNK pathways seem to be involved in histone lysine demethylase PHF8-mediated CCL7 expression in macrophages stimulated by ovarian cancer cells. The previous findings and our current data suggest that some soluble factors from cancer cells can activate the p38/JNK pathway, resulting in CCL7 overexpression via transcriptional regulation by PHF8 in macrophages. Further studies are needed to elucidate the exact factor derived from cancer cells for CCL7 induction in macrophages.

Our results demonstrated that activation of the CCL7-CCR3 axis promoted the invasion and migration of ovarian cancer cells via the ERK pathway. Similar to our results, there are reports showing that CCL7 interactions with CCR3 promote the metastasis of neurofibroma [62] and colorectal cancer [42]. CCL7 also promoted colon cancer cell metastasis via CCR3 through both the ERK and JNK pathways [30]. Considering the well-characterized role of MMP-9 and MMP-2 in the metastases of many cancers, including ovarian cancer [63], we investigated whether CCL7-induced ERK activation mediated the expression of these enzymes. The expression of MMP-9, but not MMP-2, was associated with ERK activation and cancer invasion by CCL7 in human ovarian cancer cells. In addition, we found that ERK activation and MMP-9 expression were required for ovarian cancer cell migration by CCL7 (Supplementary Figure S5).

## 4. Materials and Methods

### 4.1. Materials

Roswell Park Memorial Institute (RPMI) 1640 medium, Opti-modified Eagle’s medium (Opti-MEM), fetal bovine serum (FBS), penicillin, and streptomycin were procured from Life Technologies, Inc. (Grand Island, NY, USA). First-strand cDNA synthesis kit was procured from Amersham Pharmacia Biotech (Oakville, ON, Canada). Easy Blue^®^ for RNA isolation and protein lysis kits were purchased from Intron Biotechnology (Seoul, South Korea). Neutralizing antibody for anti-CCL7 was purchased from Abcam (Cambridge, MA, USA). Human recombinant CCL7 was obtained from Perotech (Rocky Hill, NJ, USA). CCR3 inhibitor SB297006 was purchased from B&D Life Sciences (San Diego, CA, USA). MEK inhibitor PD98059, PI3K/Akt inhibitor LY294002, JNK inhibitor SP600125, p38 inhibitor SB203580, and matrix metalloproteinase (MMP)-9 inhibitor SC311437 were obtained from Calbiochem (San Diego, CA, USA). Transwell inserts with polyvinylpyrrolidone-free polycarbonate filter (8-μm pore) and BD Matrigel™ Basement Membrane Matrix were purchased from BD Biosciences (San Jose, CA, USA).

### 4.2. Cell Cultures

Human ovarian cancer cells (A2780 and OVCAR3) and monocyte THP-1 were originally from the American Type Culture Collection. THP-1 cells were differentiated into macrophages with phorbol myristate acetate (100 nM; Sigma-Aldrich, St. Louis, MO, USA) for 24 h. Cells were incubated in a humidified atmosphere containing 5% CO_2_ at 37 °C. To acquire conditioned medium (CM) derived from human ovarian cancer cells and macrophages, the cells (2.0 × 10^6^) were cultivated into a 60 mm culture dish in 3 mL of fresh RPMI medium for 24 h. The harvested media were centrifuged for 3 min at 2400 rpm, and the supernatant (i.e., CM) was collected and deposited at −80 °C until use. As for a control, medium without cells cultivated under the same experimental conditions was prepared. THP-1-derived macrophages were activated into TAMs by incubating with the CM of A2780 or OVCAR3 cells for 24 h. We have used both A2780 and OVCAR3 cells for all the experiments in this study to avoid cell line-specific observation.

### 4.3. Invasion Assay

Matrigel™-coated synthetic basement membrane was used to determine the vitro cellular invasion. Briefly, Matrigel™ (1 µg/mL) was added to polycarbonate filters of the Transwell and allowed to form a layer for 4 h at 37 °C. Ovarian cancer cells were resuspended in plain medium (for CCL7 recombinant treatment) or CM of MQ, macrophages stimulated by A2780 cells (A-MQ), and macrophages stimulated by OVCAR3 cells (O-MQ) containing 1% fetal bovine serum, and seeded into Matrigel™-coated upper chambers. Medium containing 10% fetal bovine serum was added to the lower chamber. After a 48 h incubation, cells that invaded through the Matrigel™ to the lower surface of the membrane were fixed with methanol for 10 min and stained with 5% (*w*/*v*) crystal violet (Sigma-Aldrich) for 30 min. After removing the remaining cells from the top chamber using a cotton swab, cells on the underside of the filter were counted under an inverted microscope (Olympus, Tokyo, Japan). Cells that had invaded were counted using five randomly selected fields at ×200 magnification.

### 4.4. Cytokine Antibody Array

Cytokine profiles in the CM of MQ, A-MQ, and O-MQ were analyzed using a cytokine antibody array kit (RayBiotech, Norcross, GA, USA) following the manufacturer’s instructions. The membranes were blocked for 30 min, then incubated for 2 h with 1 mL of CM prepared from macrophages. After washing, the membranes were incubated with biotin-conjugated antibodies for 2 h and then with horseradish peroxidase-conjugated streptavidin for an additional 2 h. The signals were viewed using a chemiluminescence detection system and analyzed using Image Quant Las-4000 (GE Healthcare Life Science, Tokyo, Japan).

### 4.5. CCL7 Production

The levels of CCL7 in the conditioned medium of MQ, A-MQ, and O-MQ were measured using ELISA kit according to the manufacturer’s instructions (R&D Systems; Minneapolis, MN, USA).

### 4.6. RT-PCR

The total RNA was isolated using the Easy Blue^®^ kits, reverse transcribed into first-strand cDNA, and analyzed by PCR. The real-time RT-PCR was performed using Thermal Cycler Dice Real-Time PCR System and SYBR Premix Ex Taq™ Kit (TaKaRa, Kyoto, Japan). Thermocycler conditions for real-time PCR are as following: preincubation at 95 °C for 5 min; 40 cycles of 95 °C for 5 s, 55 °C for 10 s, and 72 °C for 20 s. Final melting curve analysis was performed to confirm the specificity of PCR reaction. The comparative CT (cycle threshold) method was used for the relative quantification of mRNA expression of each gene of interest. Gene expression was normalized with respect to β-actin. The human specific oligonucleotide primer sets were procured from Bioneer (Seoul, South Korea). All primer sequences used for the PCR analyses are listed in Supplementary Table S1. To investigate the involvement of PI3K/Akt, ERK, JNK, and p38 in the overexpression of CCL7 mRNA in OC-MQ, we used chemical inhibitors (LY294002, PD98059, SP600125, and SB203580). Initially, a range of concentrations of the inhibitors was selected based on the biological activity data such as IC50 values from manufacturers and the effective concentrations shown in previous studies [64,65,66,67,68]. After cell viability assay, the concentrations that did not induce cytotoxicity was finally selected for RT-PCR experiments.

### 4.7. Gene Expression in the Peritoneal Macrophages of Patients

To evaluate the expression of CCLs in the peritoneal macrophages from patients with ovarian cancer, we used gene expression datasets that are available from the European Bioinformatics Institute Array Express data repository (accession numbers E-MTAB-3167, E-MTAB-3398, E-MTAB-4162, E-MTAB-4764) [69]. The control group included resident peritoneal macrophages isolated from the peritoneal lavage fluids of women with non-malignant disease (*n* = 4). TAMs were isolated from the malignant ascites of untreated patients undergoing surgery for ovarian cancer (*n* = 16).

### 4.8. Transfection

To knockdown the expression of a gene of interest, cells were transiently transfected with small interfering RNAs (siRNAs) of the gene using Lipofectamine^®^ RNAiMAX (Invitrogen, Carlsbad, CA, USA) according to the manufacturer’s instructions. Briefly, cells were seeded and allowed to reach the confluency of 60–70%. Each transfection mixture prepared by mixing Lipofectamine^®^ RNAiMAX and siRNA in serum-free Opti-MEM was added to the cells and incubated for 24 h. siRNAs for CCL7, MYC, PML, and PHF8 and the control siRNA were obtained from Bioneer Technology.

### 4.9. Western Blot Analysis

The cells from different groups were lysed in protein lysis buffer (Intron Biotechnology) containing various protease inhibitor cocktail and PMSF (1 mM). After centrifugation of the lysate at 4 °C, the supernatant was collected and used for the determination of protein concentration using a Bradford assay. The cells lysates containing equal amounts of proteins (5–25 µg) was resolved using an 8–12% SDS-polyacrylamide gel electrophoresis and the proteins were electrotransferred to polyvinylidene fluoride (PVDF) membranes. The membrane was blocked with 5% skimmed milk for 30 min in Tris-buffered saline (TBS; Boster Biological Technology, Ltd., Wuhan, China), and then incubated in appropriate primary antibodies in 1% skimmed milk in TBS containing 0.1% Tween20 (TBST) overnight at 4 °C. The membrane was washed three times in TBST, and then incubated with the corresponding secondary antibodies diluted in 1% skimmed milk in TBST (1:1000–1:5000) for 1–2 h at room temperature. Enhanced chemiluminescence detection system (Amersham Pharmacia Biotech) and Image Quant Las-4000 were used for detection of antibody binding. Antibodies for β-actin, JNK, p-JNK, ERK, p-ERK, p38, MMP-9, and secondary antibodies were purchased from Santa Cruz Biotechnology (Santa Cruz, CA, USA). Anti-MMP-2 and anti-p-p38 antibodies were purchased from Cell Signaling (Beverly, MA, USA).

### 4.10. Statistical Analyses

All statistical analyses are presented as the means ± SD. Statistical analyses were carried out using a one-way analysis of variance or Student’s *t*-test, and the level of significance was set at a *p* value of < 0.05.

## 5. Conclusions

In the present study, our findings demonstrate an interaction between macrophages and ovarian cancer cells, and the potential role of this interaction in the progression of ovarian cancer. CCL7 from OC-MQs, which mimic TAMs, promoted ovarian cancer cell migration and invasion through the CCR3-ERK pathway. Consequently, our results demonstrated the pro-malignant role of the CCL7/CCR3 axis and suggested that targeting this axis could be an effective method to inhibit the metastatic spread of ovarian cancer cells by regulating the tumor microenvironment.

## Data Availability

Gene expression datasets used in this study are publicly available at the European Bioinformatics Institute Array Express data repository.

## Supplementary Materials

The following are available online at https://www.mdpi.com/article/10.3390/cancers13112745/s1, Figure S1: Expression of M2 markers in macrophages stimulated by ovarian cancer cells; Figure S2: Expression of CCL2 and CCL5 in peritoneal macrophages from patients with ovarian cancer; Figure S3: Expression and production of CCL7 in macrophages stimulated by normal ovarian surface epithelial cells; Figure S4: Involvement of CC chemokine ligand 7 (CCL7) in ovarian cancer cell invasion induced by ovarian cancer-stimulated macrophages (OC-MQ); Figure S5: Involvement of ERK signaling and MMP-9 in CCL7-induced ovarian cancer cell migration; Figure S6: Original Western Blots. Table S1: Real-time RT-PCR primer sequences.
