# Supplementary material for: CC Chemokine Ligand 7 Derived from Cancer-Stimulated Macrophages Promotes Ovarian Cancer Cell Invasion
Source: Cancers (Basel). 2021 Jun 1;13(11):2745. doi: 10.3390/cancers13112745 (PMC8198020; doi:10.3390/cancers13112745)
Supplement: Supplementary file 1 [file cancers-13-02745-s001.zip › cancers-1198244-supplementary.pdf]

Supplementary Information

Materials and Methods

Transwell migration assay

Ovarian cancer cells (1.5×10^4) were resuspended in plain medium (for CCL7 recombinant treatment) or conditioned medium of MQ, A-MQ, or O-MQ containing 1% FBS and seeded into the upper chamber of a insert. Medium containing 10% FBS were added to the lower chamber. After 24 h incubation, cells that migrated to the lower surface of the membrane were fixed with methanol for 10 min, stained with 5% (w/v) crystal violet for 30 min. After removing remaining cells from the top chamber using a cotton swab, cells on the underside of the filter were counted under an inverted microscope. Cells that had invaded were counted using five randomly selected fields at ×200 magnification.

Transcriptional regulation and conservation data analysis

To search for predicted transcription factors-binding site, we used human Chip-seq data from the Encyclopedia of DNA Elements (ENCODE) projects. Chip-seq data for 161 transcription factors with the binding motifs from Factor book repository was visualized using the UCSC genome browser (http://genome.ucsc.edu/cgi-bin/hgGateway) and inspected for the genes-binding element region. From this data, we have listed each transcription regulators conserved in the transcription factor-binding site of CCL7.

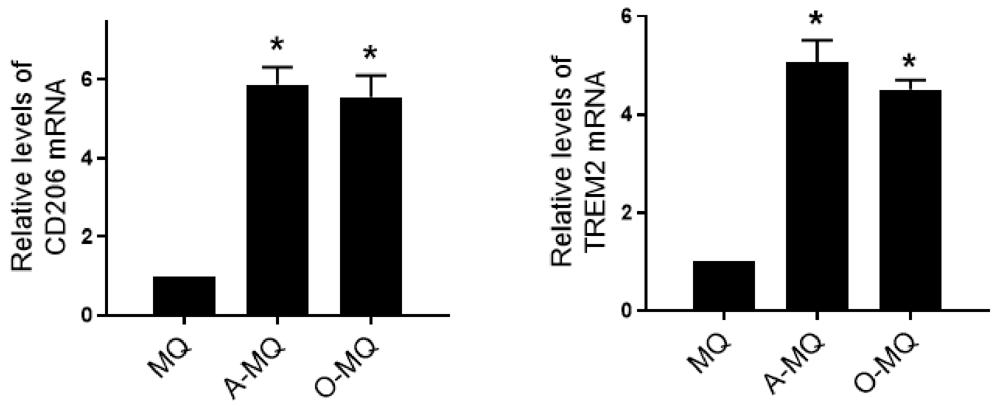

Supplementary Figure S1. Expression of M2 markers in macrophages stimulated by ovarian cancer cells

The expression of CD206 and TREM2 in MQ, A-MQ, and O-MQ were measured by RT-PCR. *$P < 0.05$.

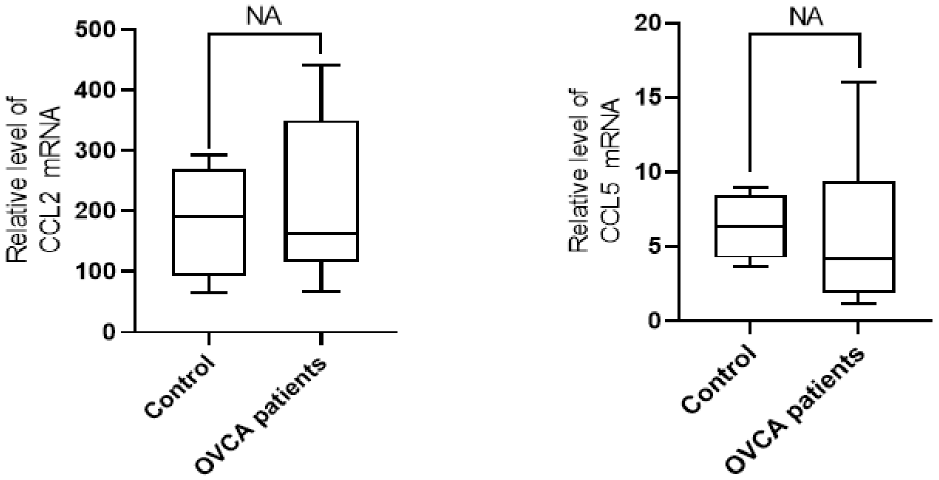

Supplementary Figure S2. Expression of CCL2 and CCL5 in peritoneal macrophages from patients with ovarian cancer

The expression of CCL2 and CCL5 in the peritoneal macrophages of women with non-malignant tumor (control) and advanced ovarian cancer (OVCA).

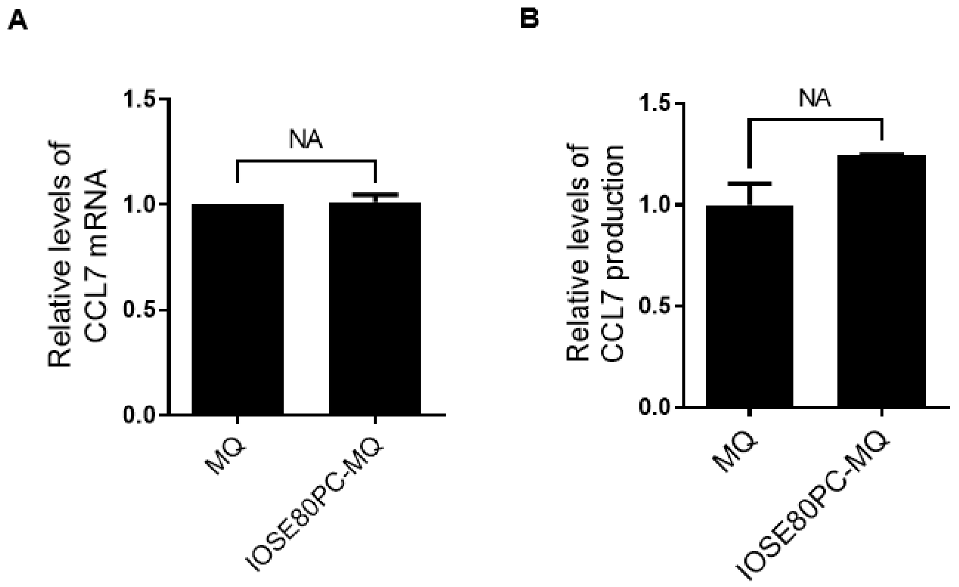

Supplementary Figure S3. Expression and production of CCL7 in macrophages stimulated by normal ovarian surface epithelial cells

(A) The expression of CCL7 in MQ and IOSE80PC-MQ were measured by RT-PCR. (B) CCL7 production from MQ and IOSE80PC-MQ was analyzed by EILSA kit.

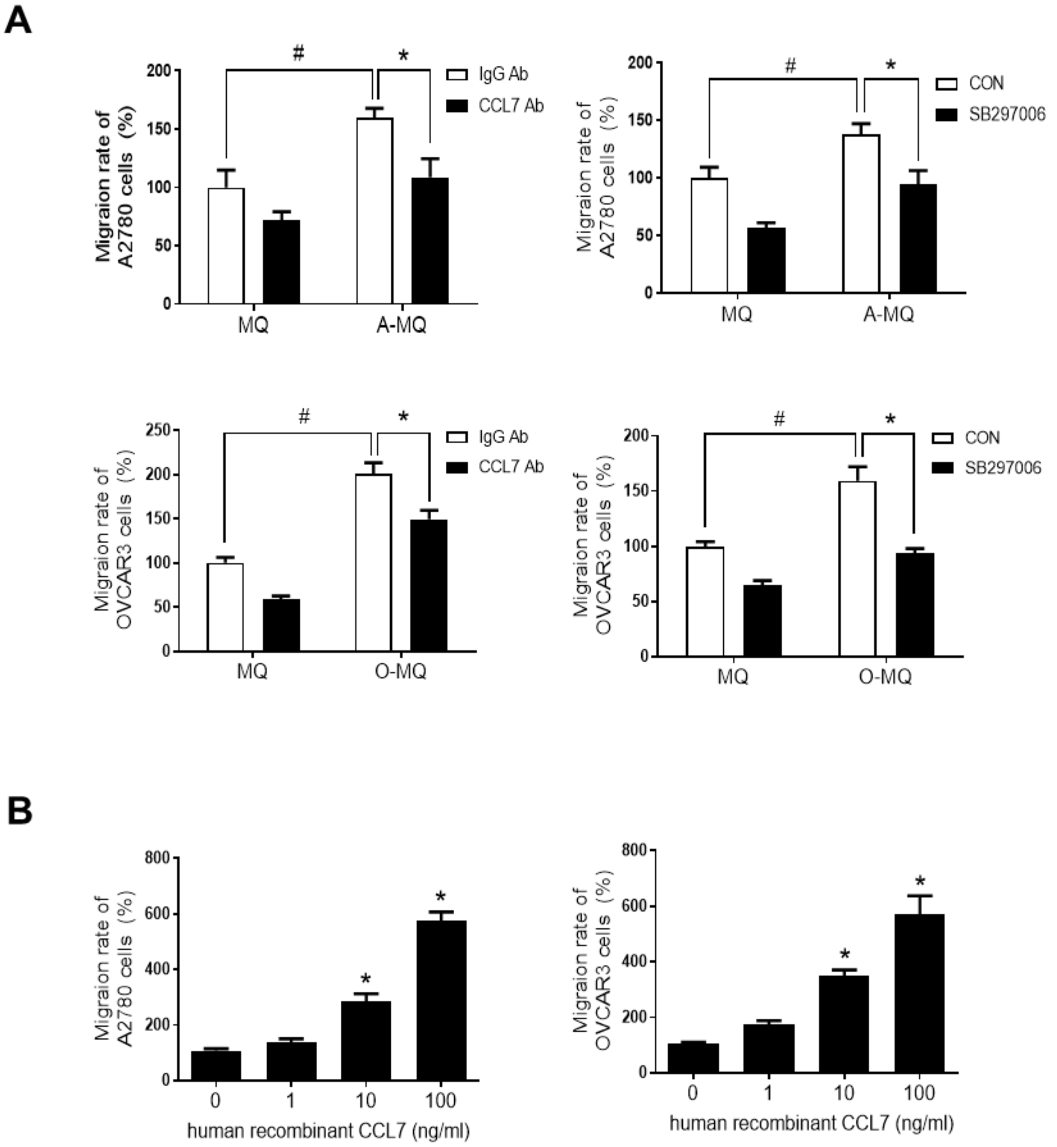

Supplementary Figure S4. Involvement of CC chemokine ligand 7 (CCL7) in ovarian cancer cell invasion induced by ovarian cancer-stimulated macrophages (OC-MQ)

(A) Human ovarian cancer cells A2780 and OVCAR3 cells were resuspended with the conditioned medium of MQ, A-MQ, and O-MQ in the absence or presence of CCL7 neutralizing antibody (3 μg/mL) and CCR3 inhibitor SB297006 (20 μM). The ovarian cancer cells were seeded in upper chambers and allowed to migrate for 24 h. (B) A2780 and OVCAR3 cells were treated with human recombinant CCL7 (0, 1, 10, 100 ng/ml) for 24 h. $^{\#,}$ $*P < 0.05$.

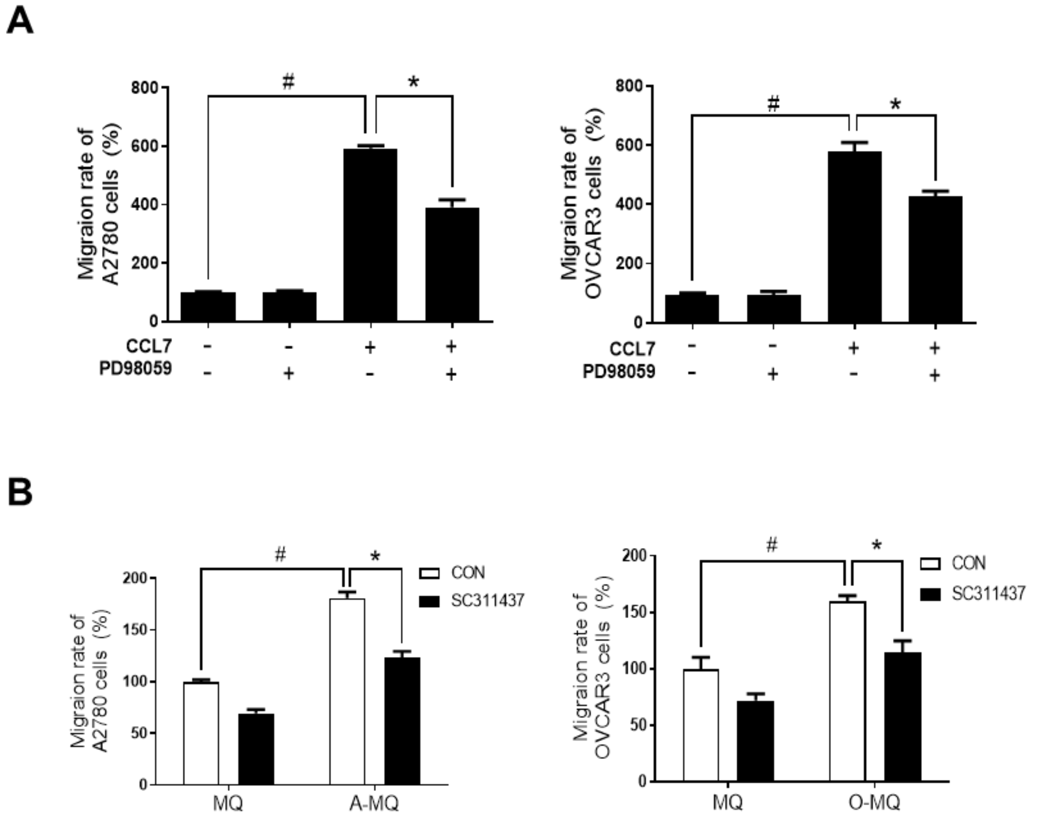

Supplementary Figure S5. Involvement of ERK signaling and MMP-9 in CCL7-induced ovarian cancer cell migration

(A) A2780 and OVCAR3 cells were pretreated with MAPK/ERK Kinase (MEK) inhibitor PD98059 (70 μM) for 1 h, and then treated with recombinant CCL7 (100 ng/ml) for 24 h. (B) A2780 and OVCAR3 cells were pretreated with MMP-9 inhibitor SC311437 (20 μM) for 1 h, and then treated with the CM of MQ, A-MQ, and O-MQ for 24 h. The results represent the mean ± SD of at least three independent experiments. $^{\#,}$ *$P < 0.05$.

Supplementary Figure S6. Original Western blots

Figure. 4B

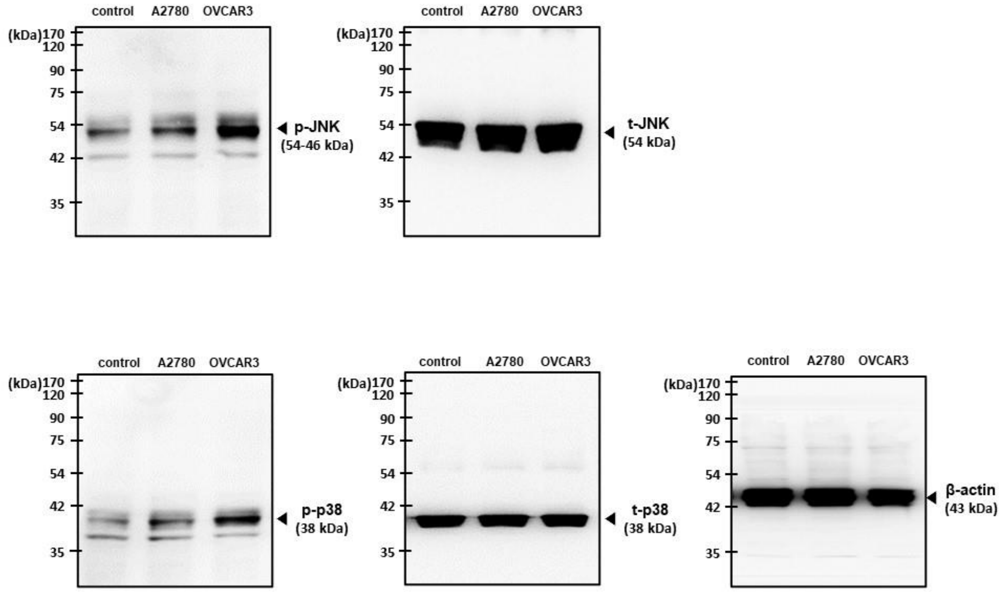

Figure. 6B

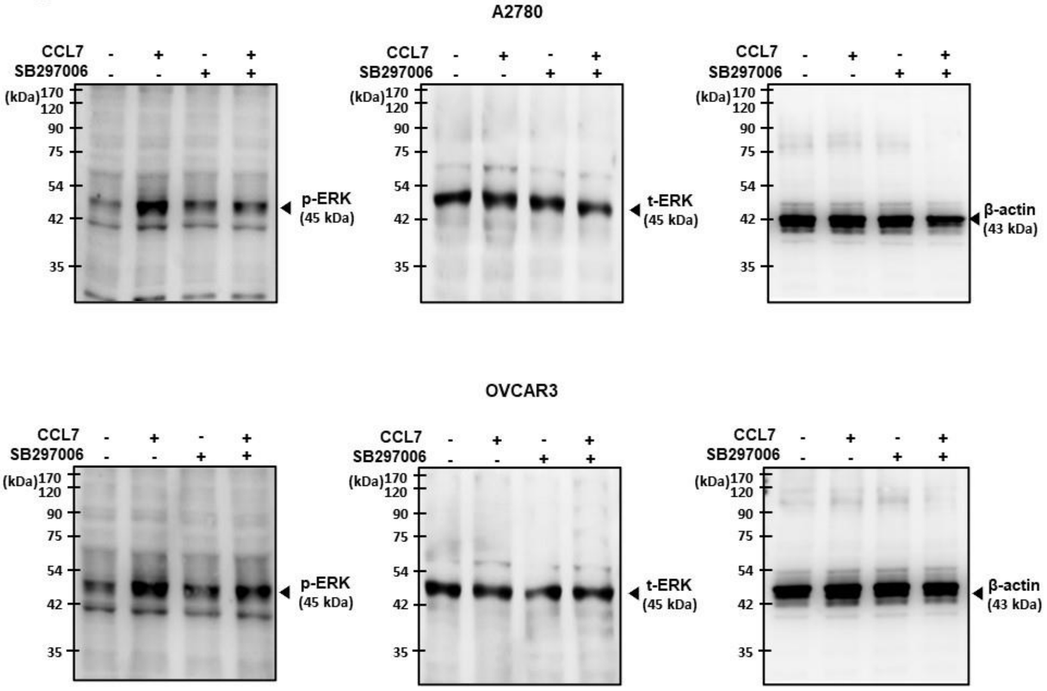

Figure. 7A

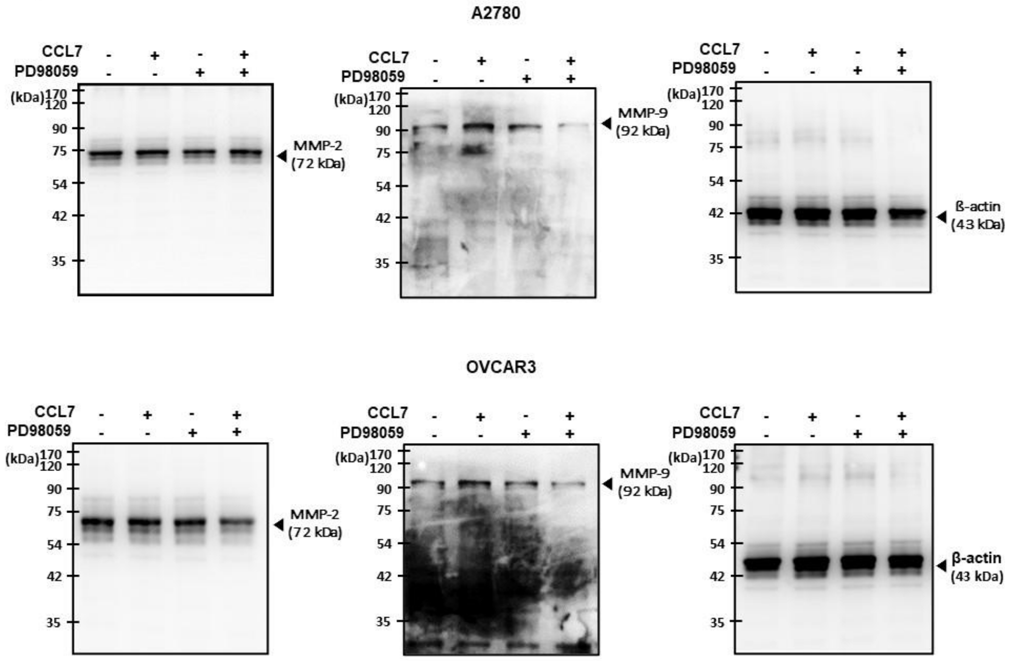
A2780
CCL7 - + - +
PD98059 - - + +
(kDa) 170 120 90 75 54 42 35
MMP-2
(72 kDa)
MMP-9
(92 kDa)
ß-actin
(43 kDa)
OVCAR3
CCL7 - + - +
PD98059 - - + +
(kDa) 170 120 90 75 54 42 35
MMP-2
(72 kDa)
MMP-9
(92 kDa)
β-actin
(43 kDa)

Supplementary Table S1. Real-time RT-PCR primer sequences

Primers	Sequences (5'→3')
CCL7	Forward: TGT ATA TGT CAT CTC AGT GCT GTA AA
	Reverse: GCT TCC ATA GGG ACA TCA TAT CTT AA
CCR1	Forward: TTT GGT GTC ATC ACC AGC AT
	Reverse: GCC TGA AAC AGC TTC CAC TC
CCR2	Forward: TGG CTG TGT TTG CTT CTG TC
	Reverse: TTC CCG AGT AGC AGA TGA CC
CCR3	Forward: TCG TTC TCC CTC TGC TCG TT
	Reverse: GCC GGA TGG CCT TGT ACT TT
MYC	Forward: CGT CCT CGG ATT CTC TGC TC
	Reverse: GCT GGT GCA TTT TCG GTT GT
PML	Forward: CGA AAA CTC GGT CTC TTC CAG
	Reverse: TGG GGA GAC CAA GTC CGA ATA
PHF	Forward: GGA ATG TTG TGG AGG AGG CT
	Reverse: TCA TCT TGC AGT CAG CCT GG
GAPDH	Forward: GAG TCA ACG GAT TTG GTC GT
	Reverse: TTG ATT TTG GAG GGA TCT CG
